# Interactive web-based lifestyle intervention and metabolic syndrome: findings from the Red Ruby (a randomized controlled trial)

**DOI:** 10.1186/s13063-015-0950-4

**Published:** 2015-09-21

**Authors:** Leila Jahangiry, Davoud Shojaeizadeh, Mahdieh Abbasalizad Farhangi, Mehdi Yaseri, Kazem Mohammad, Mahdi Najafi, Ali Montazeri

**Affiliations:** Department of Health Education and Health Promotion, School of Public Health, Tabriz University of Medical Sciences, Tabriz, Iran; Department of Health Education and Health Promotion, School of Public Health, Tehran University of Medical Sciences, Tehran, Iran; Department of Community Nutrition, Faculty of Health and Nutrition, Tabriz University of Medical Sciences, Tabriz, Iran; Department of Epidemiology and Biostatistics, School of Public Health, Tehran University of Medical Sciences, Tehran, Iran; Tehran Heart Center, Tehran University of Medical Sciences, Tehran, Iran; Mental Health Research Group, Health Metrics Research Center, Iranian Institutes for Health Sciences Research, ACECR, Tehran, Iran; Faculty of Humanity Sciences, University of Science and Culture, ACECR, Tehran, Iran

## Abstract

**Background:**

Metabolic syndrome is a growing public health problem worldwide. Several interventions have been proposed to specifically target the problem. This study evaluated the effectiveness of an interactive web-based lifestyle for management of metabolic syndrome.

**Methods:**

This randomized controlled trial was conducted from June through August 2012 in Tehran, Iran. Participants were individuals with metabolic syndrome who had registered on the study website. Interested eligible participants were invited for a free clinic visit and clinical assessment. They were randomly assigned to the intervention (*n* = 80) or control (*n* = 80) group. The intervention group received an interactive web-based program called the Healthy Heart Profile and were followed for 6 months. The control group received general information on metabolic syndrome. Anthropometric measures, glycemic status, and lipid profile were evaluated at baseline, and at 3- and 6-month follow-up assessments. Metabolic syndrome was defined according to The National Cholesterol Education Program Adult Treatment Panel (ATP) III report except for waist circumference, which was modified to ≥90 cm for both genders for the Iranian population.

**Results:**

In total, 1,437 individuals registered on the study website. The mean age of participants was 44.2 years (SD = 10.0). There were no significant differences between the intervention and control groups on any baseline variable except that participants in the intervention group recorded higher levels of LDL. The results showed a decrease in metabolic syndrome in both groups. These reductions were significantly greater in the intervention group at the 3- and 6-month follow-ups. The intervention group showed significantly greater decreases (*P* < 0.05) over the control group for, respectively, systolic blood pressure (3-month: −10 versus −6 mmHg; 6-month: −11 versus −8 mmHg), diastolic blood pressure (3-month: −10 versus −4 mmHg; 6-month: −11 versus −6 mmHg), weight (3-month: −2 versus −1 kg; 6-month: −4 versus −1 kg), body mass index (3-month: −0.5 versus −0.2 kg/m^2^; 6-month: −1.1 versus −0.4 kg/m^2^) and improvement in HDL (3-month: 2 versus 0.64 mg/dl; 6-month: 6 versus 4 mg/dl).

**Conclusion:**

The findings suggest that the web-based interactive program was beneficial for individuals with metabolic syndrome. Comprehensive interactive web-based prevention programs are promising to help involve patients in improving management of metabolic syndrome and adopting a healthy lifestyle.

**Trial registration:**

IRCT201111198132N1. Registered 27 May 2013.

**Electronic supplementary material:**

The online version of this article (doi:10.1186/s13063-015-0950-4) contains supplementary material, which is available to authorized users.

## Background

Metabolic syndrome is a growing public health problem worldwide. In Iran, it affects approximately 25 % of the population and has become a leading health concern because of its link to cardiovascular disease [1, 2]. Metabolic syndrome is identified by central obesity, increased triglycerides, reduced high-density lipoprotein cholesterol (HDL), hypertension, and elevated fasting blood glucose level [3]. The National Cholesterol Education Program Adult Treatment Panel (ATP) III defines metabolic syndrome as the presence of three or more of the following conditions: triglyceride level of at least 150 mg/dl, HDL of < 40 mg/dl in men and < 50 mg/dl in women, systolic/diastolic blood pressure (BP) of ≥ 130/85 mm Hg, fasting blood glucose (FBG) level of ≥110 mg/dl, and waist circumference > 102 cm in men and > 88 cm in women [4]. The current definitions of central adiposity are based on data from western populations; however, a growing body of literature indicates that this cut-off likely needs to be lowered for Asian populations. Several studies in Iran have suggested that a more realistic waist circumference for Iranians is > 90 cm for both genders [5–7]. ATP III states that a change in lifestyle is the essential strategy for control and management for individuals at risk of cardiovascular disease [8].

Evidence shows that lifestyle intervention programs play a crucial role in the control and treatment of metabolic syndrome. Changing dietary habits and exercise behavior can bring about a healthier waistline measurement and body mass index (BMI), improve HDL and triglyceride levels, and lower BP and blood glucose levels [9]. A recent meta-analysis of results from clinical studies on lifestyle modification and metabolic syndrome showed that the proportion of people with resolved metabolic syndrome in the intervention group was approximately twofold that of the control group. The report also showed that five out of six components of metabolic syndrome decreased significantly in the intervention groups compared to the control groups [10].

Although traditional face-to-face lifestyle intervention is the gold standard for modification of lifestyle and counseling for healthy behavior [11, 12], it faces obstacles such as availability, cost, treatment, time, travel demands, and limited effectiveness [13]. Web-based lifestyle programs can reduce these obstacles while maintaining effectiveness by offering a feasible strategy to improve health status for large numbers of at-risk people either as a stand-alone approach or as a supplement to face-to-face encounters [11, 12]. The Internet plays an increasingly important role in health care by providing a platform for large-scale delivery of information and interventions for modifying lifestyle risk factors [14].

Several review studies have explored the effectiveness of web-based and e-health intervention to improve outcomes for lifestyle modification, weight management, physical activity, and dietary intake with equivocal results [15–18]. The use of interactive web-based programs could increase the effectiveness of intervention; however, the effects of web-based interactive lifestyle modifications for people with metabolic syndrome remain unclear. The present randomized controlled trial evaluated the effectiveness of interactive web-based lifestyle intervention for metabolic syndrome. It was hypothesized that an interactive web-based educational program will promote changes in lifestyle behaviors that in turn will result in a decrease in metabolic syndrome.

## Methods

### Design overview

The Red Ruby was a randomized controlled trial conducted in Tehran, Iran. A detailed description of the method can be found elsewhere in a presentation of the study protocol [19]. Briefly, this study was designed to assess the effects of interactive web-based lifestyle intervention on metabolic syndrome. The main outcomes were a decrease in the components of metabolic syndrome. The study was concluded at the 6-month follow-up because the results indicated early effectiveness of lifestyle intervention on changing the components of metabolic syndrome [20, 21].

### Participants and inclusion criteria

A total of 160 people diagnosed with metabolic syndrome were randomly assigned to an intervention or control group. Participants who had registered on the study website (http://www.Heartresearch.ir) from June through August 2012 were recruited. These were individuals with metabolic syndrome aged 20 years and over living in Tehran and registered on the study website. The inclusion criteria were: waist circumference ≥ 90 (cut-off for metabolic syndrome in Iran for both genders [5–7]) and BP ≥ 130/85 plus one or more metabolic syndrome components, and having access to and the basic skills necessary to use the Internet. Exclusion criteria were having a history of cardiovascular disease, diabetes, cancer, or renal disease, being pregnant, taking medication for hypertension or dyslipidemia, or failing to complete the registration form. Participants were assessed at baseline and at 3- and 6-month follow-ups.

### Procedures

The study procedure from enrollment to data collection and followup assessment are present in Additiinal file 1. Announcements for recruitment were placed on virtual and non-virtual environments at the Tehran Heart Center, Tehran University of Medical Sciences, and Iranian Student News Agency. These announcements offered the opportunity to take part in a free online preventive intervention for cardiovascular disease with the aim of promoting lifestyle behavior including a healthful diet and physical activity. Those interested in participating were invited to register and complete an online form.

**Fig. 1:**
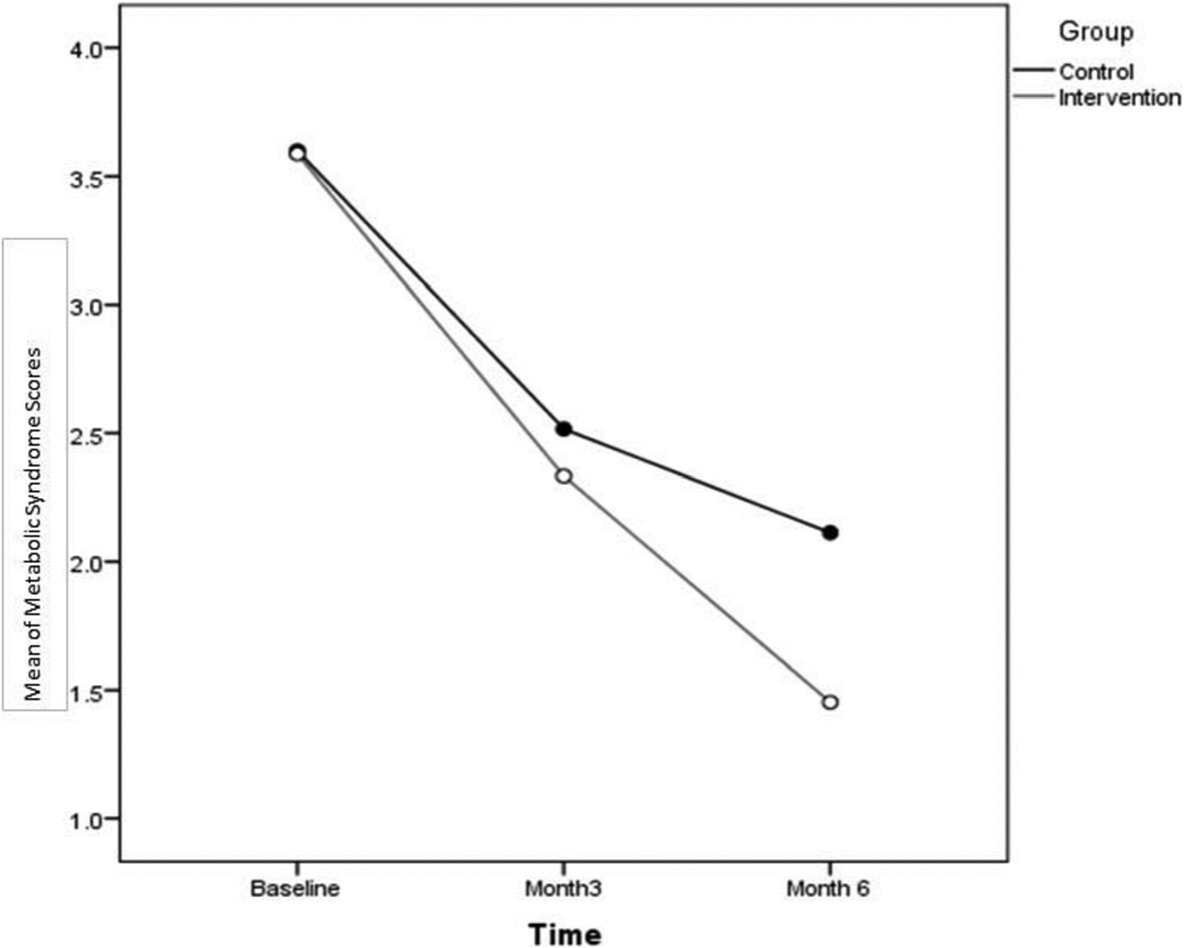
Mean values for metabolic syndrome components

The registration page included the name, gender, age, waist circumference, weight, e-mail, and address of the applicant. The homepage also explained how to measure waist circumference. The website database was reviewed by a trained research assistant to recognize registrants aged 20 and over living in Tehran (the study setting). The participants who initially enrolled were contacted by telephone for eligibility screening. During the phone interview eligible participants who were interested in participating in the study were asked to schedule a free clinic visit and clinical assessment by trained nursing staff at Tehran Heart Center.

### Randomization

The enrolled participants completed all baseline assessments and were allocated to either the intervention or control group. The allocation sequence was concealed from the main investigator (LJ) in sequentially numbered, opaque, sealed and stapled envelopes. The randomization sequence was created manually by a biostatistician using Excel software to assign participants to the study arms using a 1:1 allocation ratio with a block size of 4. Due to the nature of the study (waiting-list controlled), it was not possible to blind participants to intervention allocation.

### Intervention

Participants in both the intervention and control groups were informed of their metabolic syndrome and its components by e-mail and encouraged to make appropriate changes to their dietary intake and physical activity to manage their syndrome. The participants in the intervention group received the username and password for log-in to a personal homepage and were encouraged to regularly visit their own profile.

### My Healthy Heart Profile

My Healthy Heart Profile is a free Internet-based program with key interactive features designed for prevention of metabolic syndrome. My Healthy Heart Profile was structured into five parts:

1. Educational materials: This page was updated with new educational materials at least twice a month. The educational material included methods to lower cholesterol and control high blood pressure, conditions that put individuals at risk for cardiovascular disease, the need to lose weight, control of diabetes, the relationship between physical activity and the heart, and information on Dietary Approaches to Stop Hypertension (DASH). E-mail reminders were sent to each participant each time a new educational material was posted on the website. All users received the same educational materials. The e-mail reminders contained a brief description of the educational materials and a hyperlink to the website.
2. Personal information for name, age, gender, weight, height, telephone number, and e-mail address.
3. Inbox: This was an interactive section for exchanging messages. A calorie-restricted diet tailored by a dietitian was sent to each inbox. This diet was based on each participant’s calorie requirements according to his/her ideal body weight (IBW) and corrected body weight (CBW) where less than 30 % of the calories were derived from fat in accordance with National Heart, Lung, and Blood Institute guidelines [22]. The inbox provided an opportunity for ongoing discussion between the user and the dietitian. The users could only communicate with the dietitian through this part of the website. Users could post their answers at any time they wished and would receive a response within 24 hours.
4. Estimation of Framingham Stroke Risk (FSR) is an interactive tool by which users can calculate cardiovascular risk for 10 years based on the Persian online version of the Framingham risk score (by permission). The FSR considers the cardiovascular risk factors of age (>20 years), gender (male and female), total cholesterol (TC), HDL, systolic BP, and smoking habits. Participants can estimate their scores at every log-in and obtain feedback by text for the three traffic signals used to illustrate the three levels of risk (high, moderate, low).
5. Anthropometric and clinical measures recorded periodic measurements of user weight, waist circumference, BMI, BP, TC, LDL, HDL, triglycerides, and FBG. A simple graph displayed the measurements in one of the three warning colors (red = needs attention, orange = close to risk, green = good) for each record.

### Control

Participants in the control group were kept on a waiting list and received e-mail messages every three weeks that included information about metabolic syndrome and general information about healthy nutrition and the benefits of fruit and vegetable intake, physical activity, and lowering body weight.

### Outcome measures

The primary outcomes were a change in waist circumference, weight, systolic and diastolic BP, HDL, TC, triglycerides, LDL, and FBG. Metabolic syndrome was defined according to The National Cholesterol Education Program ATP III as the presence of three or more of the following conditions (except for waist circumference, which was set at ≥ 90 cm for both genders for Iranians): triglycerides < 150 mg/dl, HDL < 40 mg/dl for men and < 50 mg/dl for women, BP < 130/85 mm Hg, and fasting blood glucose (FBG) < 110 mg/dl.

Waist circumference was evaluated using a measuring tape to the nearest 0.1 cm. The weight of an individual dressed in light clothing without shoes was recorded each time using a calibrated scale (Seca; model 8811021658; Germany) to the nearest 0.1 kg. Height was measured without shoes using a stadiometer (Seca; Germany) to the nearest 0.1 cm. BP was measured with a mercury sphygmomanometer twice in the same arm after the individual had been seated at rest for 10–15 min. The systolic and diastolic BP measurements were the mean of the two readings. Blood sampling was collected for measurement of TC, triglycerides, LDL, HDL, and FBG for all participants. Overnight fasting for 12–14 hours was required before blood sampling. FBG was measured using the glucose oxidase method (intra- and inter-assay coefficients of variation of 2.1 % and 2.6 %, respectively). BMI was measured by using the individual’s weight in kilograms divided by the square of his/her height in meters [23].

### Sample size

The sample size was calculated based on a decrease in one standard deviation (2.5 cm) in waist circumference [24] as one of the most important components of metabolic syndrome [25]. A study with a power of 90 % at 5 % significance level requires 60 participants in each arm. Giving the risk of attrition risk, 80 participants per each group were sought.

### Statistical analysis

The data was presented as mean, standard deviation, and percentage. Normal distribution of the data was assessed using the Kolmogorov-Smirnov test and quantile-quantile (Q-Q) plots. To compare baseline variables between two groups, the *t*-test, Mann–Whitney U test, chi-square, and Fisher exact tests were employed.

Any change in scores was calculated by subtracting the baseline score from the 3- and 6-month follow-up scores. Linear mixed model (LMM) analysis was used to evaluate changes within groups. Adjustment for the multiple comparisons was done using the Bonferroni method. Time points in the analyses included baseline, during intervention (3-month follow-up), and at the termination of intervention (6-month follow-up). Differences between groups for each metabolic syndrome component and total component scores were evaluated by intent-to-treat analysis using LMM. All statistical analysis was performed by STATA (version 11.0). Results were considered statistically significant at *P* < 0.05.

### Ethics

The ethics committee of Tehran University of Medical Sciences, Vice Chancellor for Research approved the study (number 90/130/1736). Written informed consent was obtained from all participants. The CONSORT checklist is provided in Additiinal file 2.

## Results

### Participants

In total, 1,437 individuals registered on the study website. Of these, 815 records were excluded because registrants were living outside Tehran (*n* = 356), had a waist circumference < 90 cm (*n* = 392), or had incomplete information (no telephone number; *n* = 67). The remaining 622 registered participants were screened for eligibility and 305 were excluded for the following morbid conditions: cardiovascular disease (*n* = 68), diabetes (*n* = 51), consumption of antihypertensive medication (*n* = 45), consumption of cholesterol-lowering medication (*n* = 32), blood pressure < 130/85 (mmHg) (*n* = 96), renal disease or cancer (*n* = 12), or pregnancy (*n* = 1). A total of 317 individuals were invited to participate. Of these, 229 were able to attend clinical assessments and were scheduled for a baseline visit. Of the attending participants for clinical assessments, 171 met the criteria for inclusion and had metabolic syndrome. Of these, 160 participants agreed to complete the baseline measures and were randomly assigned to intervention and control groups (see Additional file 1).

The characteristics of the participants are presented in Table 1. The mean age of participants was 44.2 years (SD = 10.0) and ranged from 21 to 68 years. Participants were predominantly male (66.3 %) and married (83.8 %). Mean (SD) Internet usage was 13.2 hours per week (SD = 15.5). The mean weight was 87 kg (SD = 15) and BMI was 30.1 kg/m^2^ (SD = 4.6). There were no significant differences between the intervention and control groups on any baseline variable, except for LDL; participants in the intervention group showed higher LDL values than the control group.

**Table 1:** Baseline characteristics of study participants

	Total (*n* = 160)	Control (*n* = 80)	Intervention (*n* = 80)	*P*
Age (mean, SD)	44 .5 ± 10	44.8 ± 10	43.3 ± 10.1	0.345^a^
Gender (number, %)				0.403^c^
Males	106 (66.3)	56 (70.0)	50 (62.5)	
Females	54 (33.8)	24 (30.0)	30 (37.5)	
Education (number, %)				0.080^c^
≤12	71 (44.4)	41 (51.3)	30 (37.5)	
>12	89 (55.6)	39 (48.8)	50 (62.5)	
Marital status (number, %)				0.113^d^
Single	21 (13.1)	14 (17.5)	7 (8.8)	
Married	134 (83.8)	62 (77.5)	72 (90.0)	
Divorced/widowed	5 (3.1)	4 (5.0)	1 (1.3)	
Employment status (number, %)				>0.99^d^
Employed	159 (99.4)	79 (98.8)	80 (100.0)	
Unemployed	1 (0.6)	1 (1.3)	0 (0.0)	
Smoking (number, %)	22 (13.8)	8 (10.0)	14 (17.5)	0.168^c^
Alcohol drinking (number, %)	9 (5.7)	5 (6.3)	4 (5.1)	>0.99^d^
Drug addiction (number, %)	2 (1.3)	2 (2.5)	0 (0.0)	0.497^d^
Internet usage (hours/week) (mean, SD)	13.2 (15.5)	10.9 (13.7)	15.5 (16.9)	0.061
Weight (kg) (mean, SD)	87 ± 15	88 ± 14	87 ± 16	0.574^a^
Body mass index (kg/m^2^) (mean, SD)	30.1 ± 4.6	30.5 ± 4.5	29.8 ± 4.7	0.374^a^
Waist circumference (cm) (mean, SD)	104 ± 9	105 ± 9	103 ± 9	0.199^a^
Systolic blood pressure (mmHg) (mean, SD)	132 ± 11	132 ± 13	131 ± 8	0.593^a^
Diastolic blood pressure (mmHg) (mean, SD)	88 ± 6	88 ± 7	89 ± 6	0.594^a^
Total cholesterol (mg/dl) (mean, SD)	195 ± 39	191 ± 35	199 ± 44	0.181^a^
LDL cholesterol (mg/dl) (mean, SD)	129 ± 32	123 ± 31	134 ± 33	0.019^a^
HDL cholesterol (mg/dl) (mean, SD)				
Women	39 ± 8	39 ± 10	39 ± 7	0.683^b^
Men	46 ± 11	48 ± 12	44 ± 10	0.086^b^
Triglycerides (mg/dl) (mean, SD)	192 ± 113	198 ± 127	186 ± 96	0.534^b^
Fasting blood glucose (mg/dl) (mean, SD)	90 ± 14	91 ± 15	89 ± 12	0.772^b^
Physical activity (met/mins.) (mean, SD)	493 ± 716	471 ± 750	515 ± 684	0.224^b^

^a^Derived from *t*-test. ^b^Derived from Mann–Whitney test. ^c^Derived from chi-square test. ^d^Derived from Fisher exact test

The attrition rate in the intervention and control groups at first follow-up was the same (20 %); however, the control group had a significantly higher attrition rate (33.7 %) compared to the intervention group (20 %) at the 6-month follow-up. This information has been discussed elsewhere [26].

### Changes in metabolic syndrome indicators

Table 2 shows the distribution of baseline and follow-up values for changes in metabolic syndrome indicators over time. The results of mixed model repeated measures analysis, including the interaction between time and group, are shown. The interaction was significant for weight, BMI, systolic and diastolic BP, and HDL and indicated that the interactive web-based intervention improved metabolic syndrome components.

**Table 2:** Changes in cardiometabolic risk factors over time between groups

	Time		Control	Intervention	Time/group interaction^a^
			mean (SD)	mean (SD)	
Weight (kg)	Baseline	Value	88 ± 14	87 ± 16	<0.001
	3-month follow-up	Value	86 ± 13	85 ± 14	
		Change	−1 ± 3	−2 ± 2	
		*P* ^b^	0.059	<0.001	
	6-month follow-up	Value	87 ± 12	83 ± 15	
		Change	−1 ± 3	−4 ± 3	
		*P* ^b^	<0.001	<0.001	
Body mass index (kg/m^2^)	Baseline	Value	30.5 ± 4.5	29.8 ± 4.7	0.009
	3-month follow-up	Value	29.8 ± 3.8	29 ± 4	
		Change	−0.2 ± 1	−0.5 ± 1	
		*P* ^b^	0.079	<0.001	
	6-month follow-up	Value	29.5 ± 3.5	28.6 ± 4.4	
		Change	−0.4 ± 1	−1.1 ± 1	
		*P* ^b^	<0.001	<0.001	
Waist circumference (cm)	Baseline	Value	105 ± 9	103 ± 9	0.64
	3-month follow-up	Value	103 ± 8	101 ± 8	
		Change	−2 ± 4	−3 ± 3	
		*P* ^b^	0.385	<0.001	
	6-month follow-up	Value	100 ± 18	99 ± 9	
		Change	−5 ± 19	−4 ± 3	
		*P* ^b^	0.012	<0.001	
Systolic blood pressure (mmHg)	Baseline	Value	132 ± 13	131 ± 8	0.016
	3-month follow-up	Value	127 ± 11	121 ± 13	
		Change	−6 ± 14	−10 ± 11	
		*P* ^b^	<0.001	<0.001	
	6-month follow-up	Value	124 ± 8	120 ± 10	
		Change	−8 ± 14	−11 ± 9	
		*P* ^b^	<0.001	<0.001	
Diastolic blood pressure (mmHg)	Baseline	Value	88 ± 7	89 ± 6	0.006
	3-month follow-up	Value	84 ± 9	80 ± 9	
		Change	−4 ± 8	−10 ± 9	
		*P* ^b^	<0.001	<0.001	
	6-month follow-up	Value	82 ± 6	78 ± 6	
		Change	−6 ± 7	−11 ± 6	
		p^b^	<0.001	<0.001	
Fasting blood glucose (mg/dl)	Baseline	Value	91 ± 15	89 ± 12	0.627
	3-month follow-up	Value	89 ± 13	85 ± 10	
		Change	−1 ± 12	−4 ± 12	
		*P* ^b^	0.778	0.004	
	6-month follow-up	Value	88 ± 13	85 ± 8	
		Change	−2 ± 14	−4 ± 12	
		*P* ^b^	0.39	0.028	
Total cholesterol (mg/dl)	Baseline	Value	191 ± 35	199 ± 44	0.25
	3 months follow-up	Value	188 ± 34	186 ± 37	
		Change	−4 ± 29	−13 ± 45	
		*P* ^b^	0.409	0.019	
	6-month follow-up	Value	186 ± 28	186 ± 34	
		Change	−6 ± 24	−13 ± 41	
		*P* ^b^	0.591	0.049	
Triglycerides (mg/dl)	Baseline	Value	198 ± 127	186 ± 96	0.606
	3-month follow-up	Value	175 ± 161	160 ± 84	
		Change	−29 ± 132	−18 ± 85	
		*P* ^b^	0.075	0.04	
	6-month follow-up	Value	144 ± 68	129 ± 54	
		Change	−53 ± 82	−51 ± 77	
		*P* ^b^	0.029	<0.001	
HDL (mg/dl)	Baseline	Value	42 ± 11	41 ± 8	0.018
			0 ± 0	0 ± 0	
	3-month follow-up	Value	42 ± 12	42 ± 9	
		Change	0.64 ± 11	2 ± 9	
		*P* ^b^	0.115	0.066	
	6-month follow-up	Value	44 ± 11	46 ± 7	
		Change	4 ± 11	6 ± 8	
		*P* ^b^	0.066	<0.001	
LDL	Baseline	Value	123 ± 31	134 ± 33	0.412
	3-month follow-up	Value	118 ± 29	126 ± 33	
		Change	−6 ± 25	−6 ± 30	
		*P* ^b^	0.062	0.089	
	6-month follow-up	Value	121 ± 22	126 ± 27	
		Change	−3 ± 22	−7 ± 31	
		*P* ^b^	0.089	0.124	

^a^Based on mixed model

^b^Based on mixed model, adjusted for the multiple comparisons by Bonferroni method

Participants in both the intervention and control groups had lost a significant amount of weight by the 6-month follow-up. This was significantly greater in the intervention group. At the 6-month follow-up, the intervention group showed a significantly greater decrease (*P* < 0.001) in systolic BP (3-month: −10 versus −6 mmHg and 6-month: −11 versus −8 mmHg, respectively) and diastolic BP (3-month: −10 versus −4 mmHg and 6-month: −6 versus −11 mmHg). Significant changes in HDL and BMI (*P* < 0.05) were apparent in the intervention group during follow-up. Within-group changes were observed in both the intervention and control groups for weight, BMI, waist circumference, BP, and TG during the follow-up assessments. The within-group changes were significant in the intervention group, but not in the control group.

The overall number of participants with metabolic syndrome (showing at least three components) between groups was also assessed. There were no significant differences between groups for the number of metabolic syndrome components at baseline, but generalized LMM analysis (GLMM) at follow-up showed significant differences between groups for the number of metabolic syndrome components (*P* = 0.007; Fig. 1).

## Discussion

The findings from this web-based interactive randomized controlled trial showed that 6 months of lifestyle intervention positively influenced several metabolic syndrome components. The number of participants with metabolic syndrome decreased in both arms of the study; however, a significantly greater decrease accompanied by a lower attrition rate was observed for the intervention group. The lifestyle intervention used was an interactive education strategy that provided information and skills for participants to assist them in achieving the recommended physical activity levels and following a healthful diet plan.

Significant improvements were observed for weight loss, reduced BMI, systolic, and diastolic BP, and increased HDL at the 6-month follow-up. A similar study from Korea showed that an 8-week Internet-based cardiovascular risk reduction program resulted in significant changes in cardiovascular risk, waist circumference, diastolic blood pressure, and fasting plasma glucose among male workers with metabolic syndrome [27]. Although some studies detect no effect on cholesterol management [28], evidence from recent studies support the current findings [29–31]. Bond et al. [32] reported that a 6-month web-based intervention in addition to usual simple care increased HDL-C, and decreased weight and HbA1C in patients with type 2 diabetes mellitus. They reported that serum cholesterol and weight decreased in both the intervention and control groups; however, the decrease was greater in the intervention group (*P* < 0.05), results which are consistent with the findings of the present study.

Web-based interactive programs can motivate participants to adhere better to clinical assessments and desired behaviors for physical activity and a healthful diet. Online health assessments provide valuable feedback and facilitate opportunistic intervention by including assessment of multiple risk factors that provides a comprehensive picture for patients and encourages them to change lifestyle risk factors [31, 33].

One consistent finding on the topic is that the use of multiple behavioral change techniques significantly increased the effectiveness of a program [34–36]. The present study showed that the use of an interactive website that is frequently updated for informational content with e-mail notifications, interactive risk assessment tools, and tracking tools appeared to contribute to a change in lifestyle and had a positive effect on metabolic syndrome components. Assessing cardiovascular disease risk by using easy-to-use online risk assessment tools could facilitate better decision-making about lifestyle recommendations.

Risk factor assessment is the first step in primary prevention and guides treatment strategy because the intensity of the preventive recommendations is tailored to a patient’s level of risk [37, 38]. The mode of delivery was a key influencing factor when encouraging and persuading a participant to follow clinical assessments. Similar studies have shown that sending tailored and timely feedback, providing regular new and specific content with periodic prompts, and sending reminders result in better outcomes [39]. It appears that, in the current study, following up of participants for clinical assessment, sending the results of clinical assessments by e-mail, and recording the assessments on their profiles were additional factors to achieving successful results for metabolic syndrome management.

One surprising finding was that, although women were more likely to use the Internet to look for health information than men, in line with previous studies [40–42], men were more interested in the web-based programs and participated more than women (66.3 % versus 33.8 %). This study highlights the importance of interactive web-based programs that have shown promise for effective changes in lifestyle. However, in the battle against the epidemic of metabolic syndrome, the primary care settings are still the front lines [43].

## Conclusions

This randomized controlled trial showed the potential benefits of a web-based interactive program for individuals with metabolic syndrome. The comprehensive interactive web-based prevention program was a promising method of increasing the involvement of the participants in improved management of metabolic syndrome and adoption of a healthy lifestyle.

## Additional files

Additional file 1:
**Flow diagram of study.** Flow diagram of the study based on the CONSORT statement. (DOC 35 kb) Additional file 2:
**CONSORT checklist for study.** File contains the checklist for the study as suggested by the CONSORT statement. (DOC 206 kb)

## Abbreviations

ATP: Adult Treatment Panel
BMI: body mass index
BP: blood pressure
CBW: corrected body weight
CVD: cardiovascular disease
DASH: Dietary Approaches to Stop Hypertension
FFQ: food frequency questionnaire
FSR: Framingham Risk Score
GHQ: general health questionnaire
HDL: high-density lipoproteins
IBW: ideal body weight
IPAQ: International Physical Activity Questionnaire
LDL: low-density lipoproteins
QOL: quality of life
RCT: randomized controlled trial
TC: total cholesterol
WC: waist circumference
