# Supplementary material for: Interactive web-based lifestyle intervention and metabolic syndrome: findings from the Red Ruby (a randomized controlled trial)
Source: Trials. 2015 Sep 21;16:418. doi: 10.1186/s13063-015-0950-4 (PMC4578667; doi:10.1186/s13063-015-0950-4)
Supplement: Additional file 1: — Flow diagram of study. Flow diagram of the study based on the CONSORT statement. (DOC 35 kb) [file 13063_2015_950_MOESM1_ESM.doc]

**Enrollment**

Excluded: living outside the study setting (n = 356)

Registration on the website (n=1437)

n=1436 (male: 928, female 508)

Web-based self-report of waist circumference (n=1081)

Excluded: waist circumference less than 90 cm (n= 392), incomplete response (n=37) and without telephone number (n=30)

Telephone interview (n=622)

N=622 (Male: 478, Female: 160)

Excluded according to study criteria (n=305)

N=622 (Male: 478, Female: 160)

Invited for the study (n=317)

Excluded: not interested for more assessment (n =88)

Attended (n= 229)

Excluded: not having at least 3 components of metabolic syndrome (n=58)

Baseline measurements (n= 171)

Excluded: not interested for further participating in the study (n =11)

**Allocation**

Randomization: (n=160)

Assigned to the control group: sending e-mails every 3 weeks to visit the study website and read general information on nutrition and physical activity (n=80)

Assigned to the intervention group: interactive lifestyle intervention with Healthy Heart Profile on nutrition, and physical activity (n=80)

Lost to follow up (n=17)

Lost to follow up (n=17)

**Follow up**

3 months follow up (n=64)

measurements

Lost to follow up (n = 11)

3 months follow up (n=64)

measurements

6 months follow up (n =53)

6 months follow up (n=64)

**Analysis**
